# Roasting‐Induced Changes in the Chemical Composition, Fatty Acid Profile, and Bioactive Properties of Kaluteh Date Pits

**DOI:** 10.1002/fsn3.72479

**Published:** 2026-10-29

**Authors:** Sedighe Moghbeli, Fatemeh Heidari‐Dalfard, Elham Assadpour, Mohammad Mahdi Shojaa‐Fard, Seid Mahdi Jafari

**Affiliations:** ^1^ Department of Food Materials and Process Design Engineering Gorgan University of Agricultural Sciences and Natural Resources Gorgan Iran; ^2^ Department of Food Science and Technology, Faculty of Agriculture University of Jiroft Jiroft Iran; ^3^ Food Industry Research Co. Gorgan Iran; ^4^ Food and Bio‐Nanotech International Research Center (Fabiano) Gorgan University of Agricultural Sciences and Natural Resources Gorgan Iran; ^5^ Amir Kabir Borna Bam Company Bam Iran; ^6^ Halal Research Center of IRI, Iran Food and Drug Administration Ministry of Health and Medical Education Tehran Iran

**Keywords:** bioactive compounds, date kernel, extraction optimization, roasting treatment, waste valorization

## Abstract

Date pits (DPs), accounting for approximately 10% of the total fruit weight, are a major by‐product of the date processing industry and are generated in large quantities as waste. By containing valuable nutritional and bioactive compounds, DPs have considerable potential for utilization as functional ingredients in food applications. The present study aimed to investigate the chemical composition, fatty acid (FA) profile, and total phenolic content (TPC) of Kaluteh DPs, as well as to evaluate the effects of roasting and extraction conditions on their bioactive properties. Raw DPs contained approximately 74.92% carbohydrates, 9.30% fat, 8.45% moisture, and 6.21% protein. FA analysis revealed that lauric acid was the predominant saturated FA, whereas oleic acid was the major unsaturated FA. Response surface methodology indicated that increasing extraction temperature, extraction time, and solvent‐to‐solid ratio (SS_R_) enhanced both TPC and antioxidant activity. The optimum conditions for maximizing TPC were 63°C, 2.03 h, and a SS_R_ = 0.017 g/mL, whereas the highest antioxidant activity was achieved at 51°C, 3 h, and a SS_R_ = 0.016 g/mL. Under these optimized conditions, roasted DP extracts had a TPC = 14.8 mg GAE/g and a DPPH radical scavenging of 98.53%. Roasting also reduced moisture content to 4.05% and induced changes in FA composition, with oleic and lauric acids remaining the predominant FAs in roasted samples. Overall, roasting and extraction optimization significantly improved the bioactive characteristics and nutritional value of Kaluteh DPs, highlighting their potential as a natural source of antioxidants and functional ingredients for food applications.

## Graphical abstract

Kaluteh date pits (≈10% of fruit weight), a major processing by‐product, are rich in carbohydrates (74.92%) and fat (9.30%), with lauric and oleic acids as the predominant fatty acids. Roasting reduced moisture to 4.05% and, combined with RSM‐optimized extraction (63°C, 2.03 h, SSR 0.017 g/mL), yielded extracts with TPC = 14.8 mg GAE/g and DPPH scavenging of 98.53%. Roasted date pits are thus a promising natural source of antioxidants for functional foods.

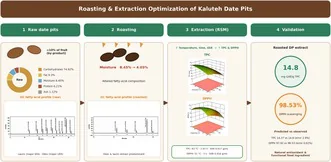

## 1 Introduction

Date palm ( *Phoenix dactylifera* L.) is one of the most important fruit crops cultivated in arid and semi‐arid regions, particularly in the Middle East and North Africa. In addition to being consumed as fresh fruit, dates are extensively processed into syrups, confectionery products, and other food ingredients, generating considerable amounts of by‐products, among which date pits (DPs) represent a significant fraction (Mahomoodally et al. 2024; Shi et al. 2023). Depending on the cultivar, DPs account for approximately 10%–15% of the total fruit weight and are generally considered agro‐industrial waste or are used as low‐value animal feed (Alkhoori et al. 2022; Zarie et al. 2023). Increasing interest in waste valorization and sustainable food production has stimulated research on the utilization of DPs as a source of value‐added compounds (Mahomoodally et al. 2024; Shi et al. 2023).

DPs are characterized by a unique chemical composition, being rich in carbohydrates, dietary fiber, lipids, proteins, minerals, and a wide range of bioactive compounds (Alkhoori et al. 2022; Mahomoodally et al. 2024). They contain considerable amounts of phenolic acids, flavonoids, tocopherols, phytosterols, and essential amino acids, which contribute to their antioxidant, antimicrobial, anti‐inflammatory, and other health‐promoting properties (Maqsood et al. 2020; Alkhoori et al. 2022). Owing to these characteristics, DPs have attracted increasing attention as promising sources of natural antioxidants and functional ingredients for food applications (Zarie et al. 2023; Mahomoodally et al. 2024). Several studies have demonstrated that the lipid fraction of DPs possesses desirable nutritional characteristics. Although the fatty acid (FA) composition varies among cultivars, oleic acid is generally the predominant unsaturated FA, whereas lauric acid is the major saturated FA (Younis et al. 2024; Alkhoori et al. 2022). Other FAs, including myristic, palmitic, stearic, and linoleic acids, are also present in appreciable amounts (Younis et al. 2024). In addition, DP oil contains phenolic compounds, carotenoids, tocopherols, and phytosterols, which contribute to its oxidative stability and functional properties, making it suitable for potential applications in food, pharmaceutical, and cosmetic industries (Mahomoodally et al. 2024; Zarie et al. 2023).

The recovery and preservation of bioactives from plant materials are strongly influenced by extraction conditions and processing treatments (Maqsood et al. 2020; Shi et al. 2025). Optimization of extraction parameters is essential for maximizing phenolic recovery and antioxidant activity (*AxAc*) (Maqsood et al. 2020). Moreover, thermal treatments such as roasting can alter the chemical composition and bioactive characteristics of plant matrices through changes in lipid composition, disruption of cellular structures, and enhanced release of bound phenolics, as well as through the formation of Maillard reaction products (Babiker et al. 2020; Shi et al. 2025). Roasting can influence the extractability and recovery of bioactive compounds from plant matrices by disrupting cellular structures and altering the accessibility of bound phenolics. For DPs, Doğanay and Baltacıoğlu (2025) reported that roasting affected the recovery of phenolic compounds and antioxidant‐related properties, demonstrating that thermal processing can modify the extractability of bioactives. These findings support the potential role of roasting as a pretreatment for improving the recovery of bioactive compounds from DPs. Despite many studies on the nutritional and functional properties of DPs, limited information is available regarding the combined effects of extraction optimization and roasting on the chemical composition, FA profile, and bioactive properties of Kaluteh DPs.

The Kaluteh cultivar was selected for the present study because of its regional importance and the availability of its pits as an agro‐industrial by‐product. Kaluteh is an important date cultivar in the southern part of Kerman province, Iran, where substantial quantities of its pits are generated during commercial date processing. Moreover, the availability of these pits through a local processing facility (Amirkabir Borna Co.) provided a practical opportunity to investigate their valorization. Compared with more extensively studied cultivars such as Mazafati and Piarom, information on the chemical composition and bioactive properties of Kaluteh DPs remains relatively limited. Therefore, the present study aimed to optimize the extraction conditions for maximizing phenolic recovery and to investigate the roasting‐induced changes in the chemical composition, FA profile, total phenolic content (TPC), and *AxAc* of DPs. The findings of this study may contribute to the valorization of this agro‐industrial by‐product and support its application as a promising source of natural antioxidants and functional ingredients in food systems.

## 2 Materials and Methods

### 2.1 Materials

Kaluteh date ( *Phoenix dactylifera* L.) pits were obtained from a local date processing factory (Amirkabir Borna Co., Bam, Kerman, Iran) during the summer harvest season, at the fully ripe (Tamar) stage. DPs were manually separated from the flesh, thoroughly cleaned to remove adhering residues, and processed within 1 week of harvest. All other chemicals and reagents used were of analytical grade and purchased from Merck (Germany) and Sigma‐Aldrich (US).

### 2.2 Preparation of Date Pit Powder (DPP)

To remove residual date flesh, DPs were thoroughly washed with tap water and cleaned. The cleaned DPs were air‐dried at room temperature for several hours and subsequently dried in a hot‐air oven at 70°C for 24 h. The dried DPs were milled using a hammer mill and passed through a 35‐mesh sieve (nominal sieve opening ≈500 μm) to obtain a uniform powder. The resulting DPP was stored refrigerated until further analyses.

### 2.3 Roasting Procedure

DPs were roasted at 180°C for 30 min using a hot‐surface heating method. For each batch, a 100 g sample was spread as a thin layer on aluminum foil (250 × 200 mm), which was placed directly on a flat stainless‐steel hot surface (70 × 50 cm) to facilitate heat transfer. The samples were manually stirred and redistributed every 2 min using a glass rod to ensure uniform roasting and minimize localized overheating. After roasting, the DPs were allowed to cool to room temperature, ground, and stored at −18°C until further analyses (Fikry et al. 2019; Paranthaman et al. 2012).

### 2.4 Proximate Composition Analysis

The proximate composition of raw and roasted DPP, including moisture, ash, crude protein, and crude fat contents, was determined according to the standard methods of the Association of Official Analytical Chemists (AOAC International 2019). Total carbohydrate content was calculated by difference.

### 2.5 Oil Extraction

Oil was extracted from raw and roasted DPP using a Soxhlet apparatus with *n*‐hexane as the solvent for 8 h, according to AOAC Official Method 920.39 (AOAC 1990). The solvent was subsequently removed using a rotary evaporator at 40°C, and the resulting oil was stored in a freezer until further analysis. Extraction yield, expressed as the percentage of oil obtained relative to the dry powder weight, was 9.84% ± 0.22% for the raw sample and 9.47% ± 0.19% for the roasted sample. Prior to GC analysis, the extracted oil was derivatized to fatty acid methyl esters (FAME) according to AOAC official methods.

### 2.6 Water Activity (*a*_*w*_) Measurement

The *a*_*w*_ of raw and roasted DPP was measured at 25°C using an *a*_*w*_ meter (Novasina AG, Lachen, Switzerland).

### 2.7 Determination of Total Phenolic Content

TPC of raw and roasted DPP was determined using the Folin–Ciocalteu colorimetric method according to Singleton and Rossi (1965). Phenolic compounds were extracted with 80% methanol under different extraction conditions of temperature, extraction time (*E*_*t*_), and solvent‐to‐solid ratio (SS_R_), as specified in Table 1. Briefly, 100 μL extract was mixed with 500 μL diluted Folin–Ciocalteu reagent (1:10, v/v) and 400 μL sodium carbonate solution (7.5%, w/v). The mixture was incubated at room temperature for 60 min, and the absorbance was measured at 765 nm using a UV–VIS spectrophotometer (UV‐1800, Shimadzu, Kyoto, Japan). TPC was quantified using a gallic acid calibration curve and expressed as milligrams of gallic acid equivalents (mg GAE)/g DPP.

**TABLE 1:** Central composite design matrix.

Treatment	Solvent‐to‐powder ratio	Temperature (°C)	Time (h)
1	0.056	45	2
2	0.1	45	2
3	0.012	25	3.5
4	0.012	45	2
5	0.1	25	3.5
6	0.056	45	0.5
7	0.1	65	0.5
8	0.1	25	0.5
9	0.056	45	3.5
10	0.056	65	2
11	0.056	45	2
12	0.056	45	2
13	0.012	65	0.5
14	0.056	25	2
15	0.012	65	3.5
16	0.012	25	0.5
17	0.1	65	3.5

### 2.8 DPPH Radical Scavenging Activity

The *AxAc* of DP extracts was evaluated using the 2,2‐diphenyl‐1‐picrylhydrazyl (DPPH) scavenging assay according to Aoshima et al. (2004). Briefly, 100 μL extract at different concentrations was mixed with 1.9 mL of methanolic DPPH solution (0.3 mM). The reaction mixture was incubated in the dark at room temperature for 20 min, and the absorbance was measured at 517 nm using a UV–VIS spectrophotometer (UV‐1800, Shimadzu, Kyoto, Japan), with methanol serving as the blank. DPPH scavenging was calculated according to Equation (1) (Mrabet et al. 2022).

(1) $$\text{DPPH scavenging activity}\;\left(\%\right)\;=100\times \frac{A_{\text{Control}}-A_{\text{Sample}}\;}{A_{\text{Control}}}$$

where *A*_control_ is the absorbance of the control and *A*_sample_ is the absorbance of the sample.

### 2.9 Fatty Acid Composition Analysis

The FA composition of raw and roasted DPP was determined by gas chromatography–flame ionization detection (GC–FID). Accordingly, FA methyl esters (FAMEs) were prepared and analyzed. Individual FAs were identified by comparing their retention times with those of a standard FA mixture, and their relative concentrations were calculated based on the corresponding peak areas. The results were expressed as the percentage of total identified FAs (%). Analyses were performed separately for raw and roasted samples to assess the effect of roasting on FA composition (Abd‐Elkarim et al. 2024).

### 2.10 Differential Scanning Calorimetry (DSC)

The thermal properties of raw and roasted DPP were evaluated using DSC according to Suresh et al. (2013). Approximately 5–10 mg of each sample was weighed into hermetically sealed aluminum pans, while an empty pan was used as the reference. The samples were heated from 90°C to 300°C at a heating rate of 5°C/min under a nitrogen atmosphere. Thermograms were recorded and used to characterize the thermal behavior of the samples.

### 2.11 Statistical Analysis

All experiments were conducted in triplicate, except for the GC and DSC analyses. Results were expressed as mean ± standard deviation. Data were analyzed by one‐way analysis of variance (ANOVA) at a significance level of *p* < 0.05. The effects of extraction temperature, *E*_*t*_, and SS_R_ on TPC and DPPH scavenging were evaluated using response surface methodology (RSM) based on a central composite design (CCD) with Design‐Expert software (Version 12, Stat‐Ease Inc., Minneapolis, MN, USA).

## 3 Results and Discussion

### 3.1 Effect of Roasting on the Proximate Composition and Water Activity

The proximate composition of DPs is presented in Table 2. Carbohydrates and crude fat were the predominant constituents, whereas moisture content (MC) was relatively low. The low MC is advantageous for storage stability, as it minimizes microbial growth and reduces the risk of quality deterioration during storage (Subhash et al. 2024). Moreover, the appreciable lipid content highlights the potential of DPs as a valuable source of edible oil and functional ingredients (Mrabet et al. 2022). Although protein content was lower than that of carbohydrates and lipids, it was within the range commonly reported for DPs, indicating their nutritional relevance. The ash content further suggests the presence of mineral constituents in the DP matrix. Overall, the results demonstrate that DPs possess a favorable nutritional profile and represent a promising source of bioactives and value‐added ingredients.

**TABLE 2:** Effect of roasting on the proximate composition and water activity of Kaluteh date pits.

Components (g/100 g (dry basis))	Before heat treatment	After heat treatment	‐VP‐value
Moisture	8.45 ± 0.21ᵃ	5.12 ± 0.18ᵇ	< 0.0001
Crude protein	6.21 ± 0.17ᵃ	6.38 ± 0.15ᵃ	0.264
Ash	1.12 ± 0.05ᵃ	1.36 ± 0.04ᵇ	0.003
Total fat	9.30 ± 0.19ᵃ	9.67 ± 0.22^a^	0.092
Total carbohydrates	74.92 ± 0.40ᵃ	77.47 ± 0.52ᵇ	0.003
Water activity	0.410 ± 0.01ᵃ	0.390 ± 0.02ᵃ	0.196

*Note:* Values are expressed as mean ± standard deviation of triplicate measurements (*n* = 3). Different superscript letters (a, b) within the same row indicate a statistically significant difference between raw and roasted samples (one‐way ANOVA, *p* < 0.05); the same letter indicates no significant difference.

These findings are consistent with previous reports. Nehdi et al. (2010) claimed that Tunisian DPs contained 10.36% oil, 5.67% protein, 72.59% carbohydrates, 1.18% ash, and 10.2% moisture. Likewise, Hossain et al. (2014) observed moisture, protein, lipid, and carbohydrate contents ranging from 3.1%–12.5%, 2.3%–6.9%, 5.0%–12.5%, and 70.9%–86.9%, respectively, on a dry weight basis (Hossain et al. 2014). Also, Mrabet et al. (2022) confirmed that DPs are rich in carbohydrates and lipids and contain appreciable amounts of proteins and minerals, supporting their potential application as functional ingredients and sustainable agro‐industrial resources.

According to Table 2, heat treatment significantly affected moisture, ash, and total carbohydrate contents (*p* < 0.05), whereas crude protein and total fat contents were not significantly affected (*p* = 0.264 and *p* = 0.092, respectively). MC decreased significantly after thermal treatment (*p* < 0.05), which could be attributed to water evaporation during roasting and the consequent increase in the relative concentration of the remaining solid components. Similar reductions in MC following roasting have been reported for DPs and other plant seeds (Babiker et al. 2020). Moreover, reductions in both MC and *a*_*w*_ induced by thermal processing have been recognized as important factors contributing to improved stability and shelf life of food products (Marcolino et al. 2024). No significant difference (*p* > 0.05) was observed in crude protein content before and after roasting, indicating the relative stability of DP proteins under the applied thermal conditions. Similar findings were reported by Babiker et al. (2020), who showed that roasting caused only minor changes in the proximate composition of DPs. Ash content increased significantly from 1.12% ± 0.05% to 1.36% ± 0.04% after heat treatment (*p* = 0.003). Since minerals are generally stable during roasting, this increase may be partly related to the reduction in moisture and the consequent increase in the relative proportion of mineral matter. Mrabet et al. (2022) also identified DPs as a source of mineral components.

The results also showed that total fat content was not significantly affected by thermal treatment, indicating the relative stability of DP lipids under the roasting conditions employed in the present study. Comparable observations were reported by Babiker et al. (2020). Furthermore, total carbohydrate content increased significantly in roasted samples, which was mainly associated with moisture reduction and the resulting increase in the relative proportion of dry matter. Similar trends have been reported for roasted plant seeds, where water loss during thermal treatment led to an apparent increase in the concentration of dry matter constituents (Al Juhaimi et al. 2024). Evaluation of *a*_*w*_ showed a numerical decrease from 0.410 ± 0.01 before heat treatment to 0.390 ± 0.02 after treatment; however, this difference was not statistically significant (*p* = 0.196). The observed reduction in *aw* may be associated with moisture loss during heating and changes in water availability within the food matrix. Similar reductions in moisture content and *aw* have been reported in roasted Baobab pits (Marcolino et al. 2024).

### 3.2 Effect of Roasting on the Fatty Acid Profile of Kaluteh Date Pit Oil

The FA profile of oil extracted from unroasted DPs revealed a mixture of saturated and unsaturated FAs, with oleic acid (24.5%) and palmitoleic acid (31.2%) as the dominant components (Table 3). The relatively high proportion of palmitoleic acid observed in the unroasted oil differs from the FA profiles commonly reported for DP oils, in which oleic acid is generally the predominant monounsaturated FA. Since FA identification in the present study was based on GC‐FID retention times, further confirmation using GC–MS and authenticated reference standards would be valuable, particularly for the identification of palmitoleic acid. Saturated FAs, including lauric (8.6%), capric (6.8%), myristic (5.5%), and palmitic acids (5.3%), were also present in appreciable amounts, while minor unsaturated FAs such as elaidic (5%) and linolelaidic acids (4.3%) contributed to the overall FA profile. These findings are generally consistent with previous studies reporting oleic acid as the major unsaturated FA and lauric acid as an important saturated FA in DP oils (Nehdi et al. 2010; Besbes et al. 2004; Hojjati 2008; Afiq et al. 2013; Ibourki et al. 2021).

**TABLE 3:** Fatty acid profile of raw and roasted date pits.

Fatty acid	Raw (%)	Roasted (%)
Caproic	6.8	—
Lauric	8.6	17.8
Capric	6.7	—
Myristic	5.5	10.9
Palmitic	5.3	10.7
Palmitoleic	31.2	—
Stearic	2.2	—
Elaidic	5	—
Oleic	24.5	52.3
Linolelaidic	4.3	8.4

*Note:* “—”: not detected/not quantified in the roasted sample.

As shown in Table 3 and Figure 1, considerable changes were observed in FA composition of Kaluteh DP oil following the roasting process. After thermal treatment, oleic acid increased markedly and reached 52.3%, indicating the predominance of monounsaturated FAs in the roasted samples. This change may be related to the relative thermal stability of oleic acid and changes in the relative proportions of individual FAs during roasting. Similar findings were reported by Babiker et al. (2020), who identified oleic acid (47.54%) as the predominant FA in roasted DPs. Likewise, Abd‐Elkarim et al. (2024) claimed that roasting and microwave treatment altered the FA composition of Siwi DPs, while oleic acid remained the major unsaturated FA. In contrast, the proportions of several saturated FAs, including lauric acid (17.8%), myristic acid (10.9%), and palmitic acid (10.7%), increased after roasting, which may reflect changes in their relative proportions following thermal processing. Moreover, linolelaidic acid content increased to 8.4% in roasted kernels compared with the unroasted samples. Mayouf et al. (2026) also reported that oleic acid and lauric acid were the predominant unsaturated and saturated FAs, respectively, in roasted DP oil. These findings are consistent with those obtained in the present study. Overall, comparison between roasted and unroasted samples indicated that thermal treatment altered the relative distribution of fatty acids, with a marked increase in the relative abundance of oleic acid. The predominance of oleic acid after roasting is of nutritional and technological importance since monounsaturated FAs have been associated with beneficial effects on cardiovascular health and enhanced oxidative stability of edible oils (Younis et al. 2024).

**FIGURE 1:**
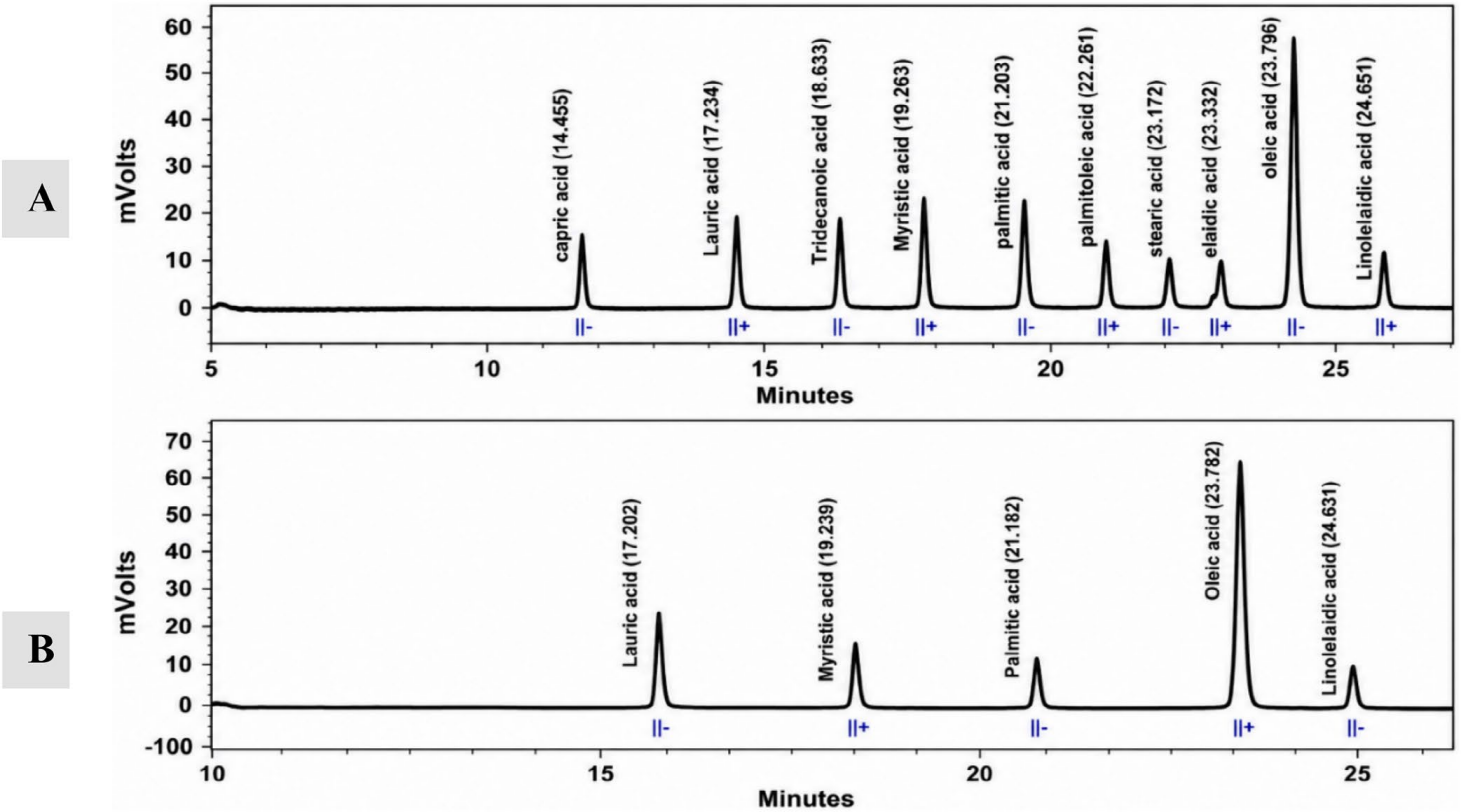
GC chromatograms of fatty acids in Kaluteh date pit oil: (A) raw, and (B) roasted sample.

### 3.3 Effect of Extraction Conditions and Roasting on Phenolic Content of Kaluteh Date Pits

As shown in Table 4, extraction temperature, *E*_*t*_, and SS_R_ significantly affected the TPC of DP extracts (*p* < 0.05). The 3D response surface and contour plots (Figure 2A–F) further illustrated the interactive effects of these process variables on phenolic recovery. Increasing extraction temperature enhanced TPC, with the highest values being obtained at elevated temperatures. This improvement can be attributed to reduced solvent viscosity, enhanced molecular mobility, improved mass transfer, and increased cell wall permeability, all of which facilitate the release of phenolics bound to the cellular matrix (Mahmud et al. 2019; Spigno et al. 2007; Ho et al. 2007). Similar findings were reported by Mrabet et al. (2022), who claimed that higher extraction temperature promoted the release of phenolics and improved the *AxAc* of DP extracts.

**TABLE 4:** ANOVA (a) and model fit statistics for the quadratic model of phenolic content (b).

Source	Sum of squares	df	Mean square	*F*‐value	*p*	Remark
**(a)**						
Model	106.93	9	11.88	157.58	< 0.0001	Significant
A‐A	60.37	1	60.37	800.66	< 0.0001	
B‐B	15.62	1	15.62	207.16	< 0.0001	
C‐C	20.02	1	20.02	265.52	< 0.0001	
AB	0.6555	1	0.6555	8.69	0.0215	
AC	0.5275	1	0.5275	7.00	0.0332	
BC	1.63	1	1.63	21.62	0.0023	
A^2^	2.46	1	2.46	32.63	0.0007	
B^2^	4.31	1	4.31	57.16	0.0001	
C^2^	1.34	1	1.34	17.77	0.0040	
Residual	0.5281	7	0.0754			
Lack of fit	0.3611	5	0.0722	0.8649	0.6135	Not significant
Pure error	0.167	2	0.0835			
Cor total	107.46	16				

Parameter	Value
**(b)**	
*R* ^2^	0.9951
Adjusted *R* ^2^	0.9888
Predicted *R* ^2^	0.979
Adeq precision	40.4
C.V. (%)	2.656
Std. dev.	0.2746
Mean	10.34

**FIGURE 2:**
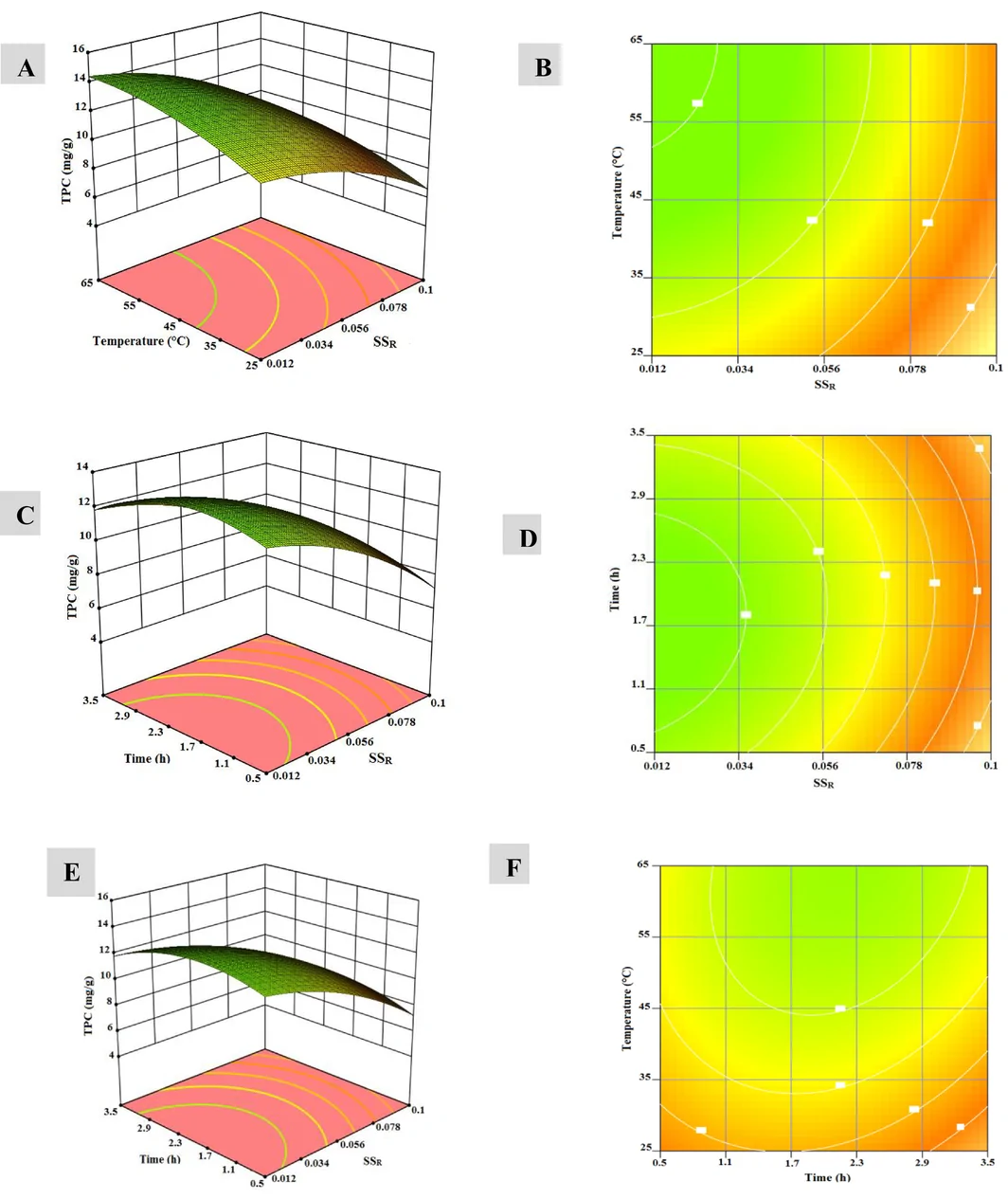
Two‐dimensional contour plots and three‐dimensional response surface plots illustrating the interactive effects of extraction variables on the total phenolic content (TPC) of date pit extracts: (A) response surface plot of temperature and solvent‐to‐solid ratio (SS_R_), (B) contour plot of temperature and SS_R_, (C) response surface plot of extraction time (*E*_*t*_) and SS_R_, (D) contour plot of *E*_*t*_ and SS_R_, (E) response surface plot of temperature and *E*_*t*_, and (F) contour plot of temperature and *E*_*t*_.

*E*_*t*_ significantly affected TPC, with prolonged contact between the solvent and the sample matrix initially enhancing mass transfer and phenolic recovery; however, extending *E*_*t*_ beyond the optimum did not result in a substantial increase in TPC. This behavior may be attributed to the attainment of extraction equilibrium and the possible oxidative degradation of certain phenolics (Chan et al. 2009). These observations are consistent with the findings of Ranasinghe et al. (2023), who reported that *E*_*t*_ contributes to the recovery of bioactives from DPs, although excessive *E*_*t*_ provides limited additional benefits.

Likewise, SSR significantly affected TPC, with higher TPC values observed at lower SS_R_. As illustrated in Figure 2A–D, the highest TPC was achieved at lower SS_R_, corresponding to higher solvent volumes. Increasing the amount of solvent enhances the contact area between the extraction medium and the plant matrix, thereby facilitating mass transfer and improving the dissolution of phenolics (Wang et al. 2016). This trend continues until equilibrium is established between the concentration of dissolved compounds and the solvent capacity. Comparable results were reported by Lucas‐González et al. (2024); higher SS_R_ improved phenolic recovery and enhanced the *AxAc* of DP extracts.

In addition to the individual effects of the extraction variables, the contour and response surface plots revealed significant interactions among the process parameters. The interaction between temperature and SS_R_ (Figure 2A,B) indicated that simultaneous increases in both variables progressively enhanced TPC. Similarly, the combined effect of temperature and *E*_*t*_ (Figure 2E,F) showed that the highest TPC was obtained at relatively high temperatures and intermediate to prolonged *E*_*t*_. Furthermore, the elliptical contour patterns and the curvature of the response surfaces confirmed the existence of interaction effects and quadratic behavior, supporting the adequacy of the second‐order polynomial model employed in RSM. Previous studies have also emphasized the importance of simultaneous optimization of extraction conditions. For instance, Shi et al. (2023) reported that extraction parameters markedly affected the TPC and *AxAc* of DP extracts, and that appropriate selection of process variables significantly improved the recovery of bioactives. Similarly, Djaoudene et al. (2024) identified temperature, *E*_*t*_, and SS_R_ as key factors governing the recovery of phenolics and the *AxAc* of DPs. Therefore, the results obtained in the present study are in accordance with previous findings and further confirm that the simultaneous optimization of extraction variables using CCD and RSM plays a crucial role in maximizing phenolic recovery and improving the accessibility of bioactives from DPs.

### 3.4 Effect of Extraction on the Antioxidant Activity of Kaluteh Date Pits

The results analyzed by CCD revealed that temperature and SS_R_ significantly influenced the *AxAc* of DP extracts, whereas *E*_*t*_ showed no statistically significant effect (Table 5). Increasing the extraction temperature from 25 to 45 enhanced the *AxAc* (Figure 3), which could be attributed to improved solvent penetration, disruption of the cell wall matrix, and increased release of phenolics. However, a further increase to 65°C resulted in a slight decline in *AxAc*, which may be associated with the thermal degradation and reduced stability of heat‐sensitive phenolics (Mrabet et al. 2022).

**TABLE 5:** ANOVA (a) and model fit statistics for the quadratic model of antioxidant activity (b).

Source	Sum of squares	df	Mean square	*F*‐value	*p*	Remark
**(a)**						
Model	1390.75	9	154.19	55.81	< 0.0001	Significant
A‐A	984.79	1	984.79	347.82	< 0.0001	
B‐B	7.25	1	7.25	2.62	0.1494	
C‐C	30.24	1	30.24	10.94	0.0130	
AB	0.1378	1	0.1378	0.0499	0.8297	
AC	15.98	1	15.98	5.78	0.0473	
BC	1.42	1	1.42	0.5138	0.4967	
A^2^	24.19	1	24.19	8.75	0.0211	
B^2^	3.78	1	3.78	1.37	0.2805	
C^2^	310.52	1	310.52	112.38	< 0.0001	
Residual	19.34	7	2.76			
Lack of fit	14.42	5	2.88	1.17	0.5201	Not significant
Pure error	4.92	2	2.46			
Cor total	1410.09	16				

Parameter	Value
**(b)**	
*R* ^2^	0.9863
Adjusted *R* ^2^	0.9686
Predicted *R* ^2^	0.9032
Adeq precision	24.6168
C.V. (%)	1.96
Std. dev.	1.66
Mean	84.82

**FIGURE 3:**
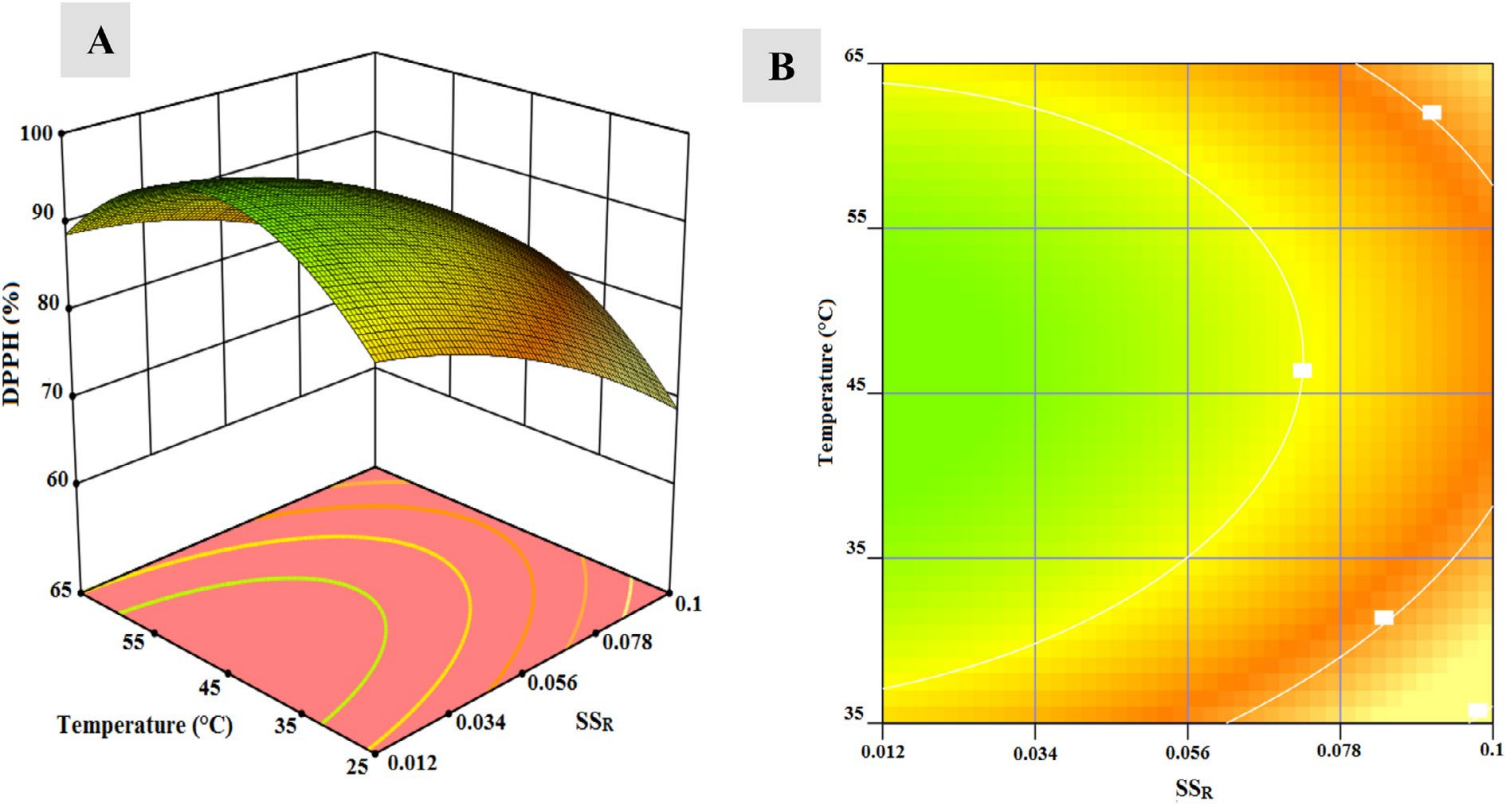
(A) Response surface plot and (B) contour plot showing the combined effects of temperature and solvent‐to‐solid ratio on DPPH scavenging of date pit extracts.

Although slight variations in *AxAc* were observed over the *E*_*t*_ investigated, ANOVA showed that these differences were not statistically significant. Therefore, within the studied range, *E*_*t*_ did not exert a considerable effect on the *AxAc* of the extracts. On the other hand, increasing the SS_R_ promoted mass transfer and facilitated the extraction of phenolic and flavonoid compounds, thereby enhancing free radical scavenging (Figure 3). Similar observations have been reported for DP by‐products, where optimization of extraction conditions improved the recovery of bioactives and *AxAc* (Zarie et al. 2023). Overall, the findings of the present study were consistent with previous reports on DP extraction. The observed trend indicated that optimization of extraction conditions, particularly temperature and SS_R_, plays an important role in enhancing *AxAc* (Al‐Farsi and Lee 2008; Afifi et al. 2017; Mrabet et al. 2022; Zarie et al. 2023).

### 3.5 Optimum Extraction Conditions and Effect of Roasting on Phenolic Content/Antioxidant Activity

The response surface methodology was used to establish mathematical models describing the effects of the extraction variables on *AxAc* and TPC. The predicted responses were expressed as functions of the coded independent variables, where A, B, and C represent SS_R_, *E*_*t*_, and extraction temperature, respectively. The fitted quadratic model for *AxAc* was expressed as Equation (2):

(2) $$\begin{array}{cc} \text{R1}=\; & 92.44-8.28A+0.82B+1.74C-0.13\mathrm{AB}\\ & +1.27\;\mathrm{AC}+0.43\mathrm{BC}-2.92A^{2}-0.11B^{2}-10.35C^{2} \end{array}$$

where R1 represents the predicted DPPH radical‐scavenging activity (%). For TPC, the fitted quadratic model was expressed as Equation (3):

(3) $$\begin{array}{cc} Y=\; & 12.06–2.46A-1.25B+1.42C+0.2863\mathrm{AB}\\ & -0.2568\mathrm{AC}+0.4513\mathrm{BC}-0.9580A^{2}-1.27B^{2}-0.7080C^{2} \end{array}$$

where *Y* represents the predicted TPC (mg GAE/g). The linear, interaction, and quadratic terms in these equations describe the individual and combined effects of the extraction variables on the measured responses.

The optimum extraction conditions for maximizing *AxAc* were determined by RSM: SS_R_ = 0.016 g/mL, an *E*_*t*_ = 3 h, and an extraction temperature of 51°C, with a desirability value = 1.000. Under these conditions, the highest DPPH scavenging was predicted. In contrast, the optimum conditions for TPC were: SS_R_ = 0.017 g/mL, an *E*_*t*_ = 2.03 h, and an extraction temperature of 63°C, also with a desirability value = 1.000. Following the determination of the optimum extraction conditions, roasted DPs were subjected to extraction under the respective optimized conditions, and the TPC and *AxAc* of the obtained extracts were evaluated. The results revealed that TPC of the roasted DP extract reached 14.8 mg GAE/g, while DPPH scavenging increased to 98.53%, indicating that the roasting process positively influenced the release and recovery of phenolics and enhanced the *AxAc* of the extracts. The close agreement between the predicted and observed values, with percentage errors of 2.9% for TPC and 0.62% for *AxAc* (Table 6), confirmed the adequacy and reliability of the RSM models for predicting the optimum extraction conditions.

**TABLE 6:** Validation of the predicted optimum extraction conditions.

Response	Predicted value	Observed value	% Error
TPC (mg GAE/g)	14.37	14.8	2.9
*AxAc* (%)	97.92	98.53	0.62

The increase in TPC and *AxAc* after roasting may be attributed to the partial disruption of cellular structures, enhanced accessibility of bound phenolics, and the formation of low‐molecular‐weight compounds with reducing properties through Maillard reactions. Thermal treatment has been reported to facilitate the release of phenolics bound to the food matrix, thereby improving their extractability and *AxAc* (Liu et al. 2020). Similar findings were reported by Babiker et al. (2020), who claimed that roasting altered the phenolic profile and enhanced the functional properties of DPs. In their study, roasted DPs exhibited TPC from 525.35 to 595.83 mg GAE/100 g, confirming the positive effect of roasting on phenolic recovery. Furthermore, Shi et al. (2025) reported that roasting intensity significantly influenced the polyphenol composition and *AxAc* of DP beverages, highlighting the important role of thermal processing in modulating the bioactive characteristics of DP products. Likewise, Peng et al. (2021) showed that roasting enhanced free radical scavenging through the release of bioactives and the formation of Maillard reaction products. Similar observations were reported by Chaikham et al. (2026), who found that roasting intensity significantly improved the *AxAc* of Sacha inchi (*Plukenetia volubilis* L.) seeds. Therefore, the findings of the present study are in agreement with previous reports and further confirm the beneficial effect of roasting on enhancing the phenolic recovery and *AxAc* of DP extracts, thereby increasing their potential application as natural antioxidants and functional food ingredients. Temperature (°C).

### 3.6 Thermal Analysis

Based on the DSC thermogram presented in Figure 4, the thermal behavior of roasted DPP revealed no discernible glass transition temperature (*T*_g_) within the investigated temperature range. The absence of a detectable *T*_g_ may be attributed to the complex composition of the DP matrix and the relatively low proportion of amorphous components detectable within the investigated temperature range. Similarly, Rahman et al. (2007) reported the absence of a *T*_g_ in DPP and attributed this behavior to its complex structure and the presence of stable crystalline constituents. Besides, Mrabet et al. (2022) described DPs as a complex matrix composed of lipids, polysaccharides, dietary fiber, and phenolic compounds, which may give rise to intricate thermal behavior.

**FIGURE 4:**
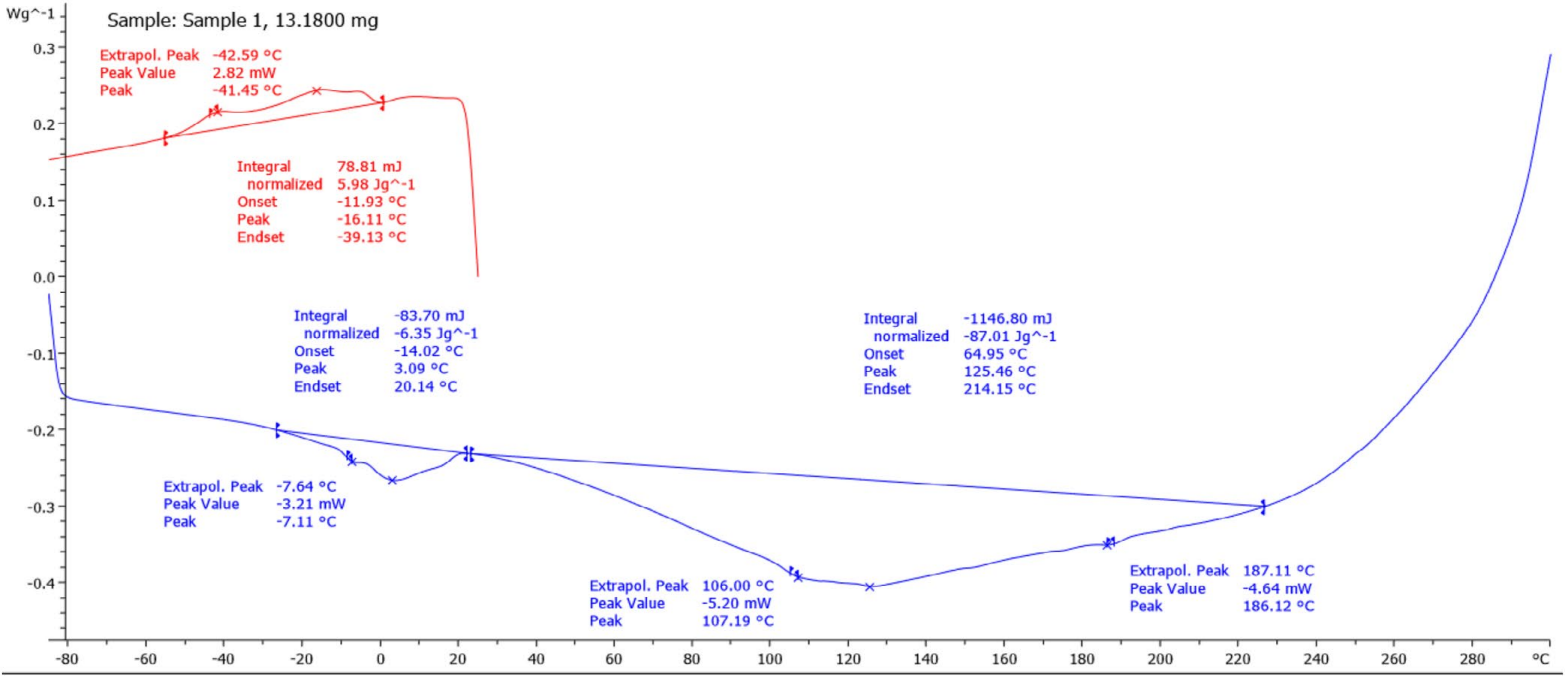
DSC thermogram of roasted Kaluteh date pit powder.

As shown in Figure 4, the DSC curve exhibited two major thermal events. The first thermal transition occurred in the range of approximately −14°C to 20°C, with a peak around 3°C. This peak is likely associated with the melting and/or rearrangement of low‐melting lipid fractions. The presence of this transition indicates the existence of lipid components undergoing phase changes at low temperatures. Similar observations were reported by Suresh et al. (2013), who identified a low‐temperature peak in the DSC thermogram of DPP and attributed it to the oil fractions. In addition, Babiker et al. (2020) demonstrated that roasting altered the FA composition and bioactives of DPs, changes that may consequently influence their thermal properties. The second thermal event was observed over 64.95°C–214.15°C, with a maximum peak at approximately 125.46°C. The broad nature of this peak suggests that multiple thermal phenomena occurred simultaneously. This region is most likely associated with the melting of more thermally stable lipid fractions, together with thermal transitions of non‐lipid constituents present in DP matrix. Suresh et al. (2013) likewise attributed the second peak to non‐lipid components and suggested that the coexistence of compounds with different thermal stabilities results in broad transition regions in DSC thermograms. Besides, Mrabet et al. (2022) reported that the complex chemical composition of DPs and the presence of phenolics, dietary fiber, and other bioactives could lead to multistep thermal behavior. It should be noted that changes in thermal behavior do not necessarily imply substantial changes in the overall chemical or proximate composition of the sample. DSC is sensitive to the physical state, phase transitions, molecular interactions, and organization of different components within the matrix, which may change during roasting without causing large changes in the measured proximate composition. Therefore, the differences observed in the DSC thermogram may reflect changes in the physical organization and intermolecular interactions of lipid and non‐lipid constituents induced by roasting rather than major changes in their overall amounts (Suresh et al. 2013).

Overall, the DSC results revealed distinct thermal transitions in roasted DPP, reflecting the complex thermal behavior of its lipid and non‐lipid constituents. The absence of a detectable *T*_g_ and the presence of two distinct thermal regions indicate the complex thermal behavior of roasted DPP, which may be substantially influenced by lipid components and their interactions with other constituents of the matrix. However, the absence of *T*_g_ alone does not provide sufficient evidence to confirm the crystalline or amorphous nature of the sample. These findings are in agreement with those reported by Rahman et al. (2007), Suresh et al. (2013), Babiker et al. (2020), and Mrabet et al. (2022), highlighting the important role of chemical composition and roasting treatment in determining the thermal characteristics of DPs.

## 4 Limitations of the Study

Several limitations of the present study should be acknowledged. The fatty acid profile was determined using GC‐FID with a single analysis for each sample; therefore, replicate analyses in future studies would allow analytical variability to be assessed and statistical comparisons to be performed. In addition, GC–MS analysis in combination with authentic standards is recommended to further confirm the identification and fatty acid composition of the detected compounds. Antioxidant activity was evaluated using the DPPH assay only; incorporating complementary antioxidant assays in future work could provide a more comprehensive evaluation of antioxidant capacity. Individual phenolic compounds were not identified in the present study, and HPLC or LC–MS analysis could provide more detailed characterization of the phenolic profile. Some RSM plots were relatively flat; thus, refinement or expansion of the experimental domain may improve the identification of optimum conditions. Complementary techniques, such as SEM and FTIR, could also provide further insight into roasting‐induced structural changes. Finally, future studies should also evaluate the stability, safety, and practical performance of the optimized extracts and roasted DPP in relevant food and industrial applications.

## 5 Conclusion

In this study, the physicochemical characteristics, FA profile, thermal behavior, and bioactive properties of Kaluteh DPP were investigated before and after roasting. The compositional analysis revealed that DPs contain appreciable amounts of carbohydrates, lipids, and proteins, while roasting induced significant changes in MC, ash content, and *a*_*w*_. FA profiling demonstrated that DP oil is rich in unsaturated FAs, particularly oleic acid, which contributes to its nutritional value and oxidative stability. Thermal analysis further confirmed that roasting influences the thermal behavior of DPP. RSM successfully identified the optimum extraction conditions for maximizing TPC and *AxAc*. The optimum conditions for TPC were 63°C, 2.03 h, and a SS_R_ = 0.017 g/mL, whereas the highest *AxAc* was obtained at 51°C, 3 h, and a SS_R_ = 0.016 g/mL. Under these optimized conditions, roasting significantly enhanced the TPC and *AxAc* of the extracts, indicating improved accessibility and recovery of bioactives. The increase in *AxAc* was positively associated with the elevated TPC, highlighting the important role of phenolics in free radical scavenging. Overall, the findings demonstrate that roasted DPs represent a valuable source of phenolics, natural antioxidants, and high‐quality oil. Therefore, this abundant agro‐industrial by‐product has considerable potential for utilization as an inexpensive and sustainable raw material for the development of functional foods, nutraceuticals, pharmaceutical formulations, and cosmetic products. In practical terms, roasted DP powder could be used as a functional ingredient in bakery and cereal‐based products, potentially contributing to increased dietary fiber and antioxidant content. In addition, the phenolic‐rich extracts obtained under the optimized extraction conditions have potential for use as natural sources of antioxidant compounds in food formulations, given their higher phenolic content and antioxidant activity. Furthermore, given its favorable fatty acid profile and oxidative stability, DP oil may have potential applications in cosmetic formulations and nutraceutical products. Nevertheless, further studies are required to characterize individual bioactive compounds and to evaluate their functional properties, safety, stability, and potential industrial applications in greater detail.

## Author Contributions

**Sedighe Moghbeli:** investigation, writing – original draft, methodology, data curation. **Fatemeh Heidari‐Dalfard:** writing – original draft, methodology, formal analysis. **Elham Assadpour:** validation, formal analysis, writing – review and editing. **Mohammad Mahdi Shojaa‐Fard:** resources, funding acquisition. **Seid Mahdi Jafari:** conceptualization, writing – review and editing, supervision, visualization, project administration, formal analysis.

## Funding

The authors have nothing to report.

## Conflicts of Interest

The authors declare no conflicts of interest.

## Data Availability

The authors have nothing to report.
